# Automated classification of benign paroxysmal positional vertigo from video-nystagmography using a delay-aware neural network

**DOI:** 10.1038/s41598-026-52908-7

**Published:** 2026-05-18

**Authors:** Kunal Chaturvedi, Nicholas Yang, Imelda Hannigan, Nicole Reid, Andrew Bradshaw, Karthick Thiyagarajan, Tony Jan, Ali Braytee, Miriam S. Welgampola, Mukesh Prasad

**Affiliations:** 1 School of Computer Science, Faculty of Engineering and Information Technology, University of Technology Sydney, Sydney, Australia; 2 Central Clinical School, University of Sydney, Sydney, Australia; 3 Institute of Clinical Neurosciences, Royal Prince Alfred Hospital, Sydney, Australia; 4 School of Engineering, Design and Built Environment, Western Sydney University, Sydney, Australia; 5 Centre for Artificial Intelligence and Optimization, Torrens University, Sydney, Australia

**Keywords:** Video-nystagmography, Benign paroxysmal positional vertigo, Time-series classification, Explainable AI, Computational biology and bioinformatics, Diseases, Health care, Medical research, Neurology

## Abstract

Nystagmus is a key indicator of vestibular disorders, including benign paroxysmal positional vertigo (BPPV). Accurate diagnosis of BPPV is essential, as it is treatable with specific bedside maneuvers that lead to rapid symptom resolution, thereby improving patient outcomes and reducing unnecessary treatments. In clinical practice, identification of positional nystagmus relies on eliciting and interpreting eye movements during provocative maneuvers, with or without video nystagmography (VNG). This process can be subjective and difficult to standardize when signals are subtle, noisy, or temporally variable. We present DSF-BPPVNet, a delay-aware neural architecture for BPPV classification from VNG traces. The model combines temporal convolution, delayed-state feedback, and residual refinement to support classification from temporally structured eye-movement signals. The model was evaluated on 3,111 VNG traces from 705 patients using 5-fold cross-validation and compared with established deep-learning baselines. In the patient-independent setting, DSF-BPPVNet achieved the strongest overall performance among the evaluated models, with an F1-score of 0.819 ± 0.020. Explainability analyses were also performed to characterize model attribution patterns and temporal weighting behavior.

## Introduction

Vertigo is a common, disabling, and treatable symptom that arises from dysfunction of the peripheral vestibular organs or their central connections^1^. It has substantial effects on physical and mental health, quality of life, and daily functioning, and is associated with an increased risk of falls and injury^2^. Benign paroxysmal positional vertigo (BPPV) is the most prevalent form of recurrent vertigo, accounting for nearly 20–30% of all cases^3^. BPPV arises when displaced otoconia abnormally stimulate the semicircular canals during head movement, provoking transient vertigo together with characteristic positional nystagmus^4^. In clinical practice, diagnosis depends on eliciting and interpreting these eye movements during provocative maneuvers such as the Dix–Hallpike and roll tests, because the direction and temporal evolution of the elicited eye movements contribute importantly to clinical interpretation.

This dependence on eye-movement interpretation creates an important clinical bottleneck. Recognition of positional nystagmus remains operator-dependent, and diagnostic accuracy can vary with experience, recording quality, and testing conditions. These limitations have motivated growing interest in technology-assisted vestibular assessment, including video-nystagmography (VNG) and AI-supported interpretation. However, the value of such approaches still rests on one central requirement: reliable automated analysis of noisy VNG recordings while preserving the temporal structure used in clinical decision-making.

Clinical interpretation of positional nystagmus relies not only on movement direction but also on temporal descriptors such as latency, duration, and progression over time. These temporal patterns are clinically informative but may vary across canal involvement, debris location, and the biomechanics of head position relative to gravity^5,6^. Quantitative analysis can further summarize these dynamics using measures such as slow-phase velocity (SPV), from which features including onset delay, peak response, decay behavior, and episode duration may be derived^7–10^. Traditional automated approaches have used such handcrafted descriptors for classification^11,12^, but their performance depends on accurate feature extraction and expert-defined representations. In this work, rather than using explicitly computed SPV signals as input, we learn directly from the recorded horizontal and vertical eye-position traces so that temporal features can be inferred from the trajectories themselves.

Recent learning-based approaches have demonstrated that nystagmus analysis from recorded eye movements is feasible. Prior work has explored convolutional models^13–15^, recurrent and hybrid CNN–RNN architectures^16,17^, and more recent neural ODE-based^18^ and Transformer-based approaches^19^ for nystagmus detection and BPPV-related classification tasks. At the same time, the field has begun to expand beyond isolated backbone comparisons toward broader AI-assisted diagnostic workflows^20,21^. Despite these advances, important challenges remain. Positional nystagmus remains a temporally structured signal in which diagnostically relevant evidence may be distributed across separated segments of the trace. Standard temporal convolutions are effective at extracting local patterns, but they do not retain prior latent states as explicitly reusable representations. Recurrent formulations can aggregate prior context, yet that history is typically compressed into a hidden representation rather than maintained as a structured set of temporally separated states that can be revisited during processing. Continuous-time formulations offer a different view of temporal evolution, although their smooth latent dynamics may be less directly matched to signals containing short-lived, phase-dependent transitions and abrupt resets. In addition, once intermediate features are formed, later evidence may still help disambiguate weak responses, onset patterns, or artifact-contaminated segments.

Motivated by this problem, we propose DSF-BPPVNet, a neural architecture for BPPV classification from VNG traces that integrates delayed-state feedback, temporal convolution, and residual refinement. The central idea is to preserve prior latent states in a structured buffer and reintroduce them into the current computation through learnable weighting, allowing the model to reuse temporally separated information when it becomes diagnostically relevant. Residual refinement complements this mechanism by progressively updating intermediate representations rather than passing them forward unchanged. This is intended to improve representation quality in the presence of variability in onset, amplitude, duration, and waveform morphology. We evaluate this approach against established deep-learning baselines and further examine its behavior using ablation and attribution analyses.

The main contributions of the paper are as follows:

- DSF-BPPVNet is proposed for BPPV classification from video nystagmography, combining delayed-state feedback, temporal convolution, and residual refinement.
- The proposed model is evaluated against representative deep-learning baselines for BPPV classification.
- We use attribution-based analyses together with examination of the learned delay-weight distributions to characterize the model’s input sensitivity and internal temporal weighting patterns

**Fig. 1:**
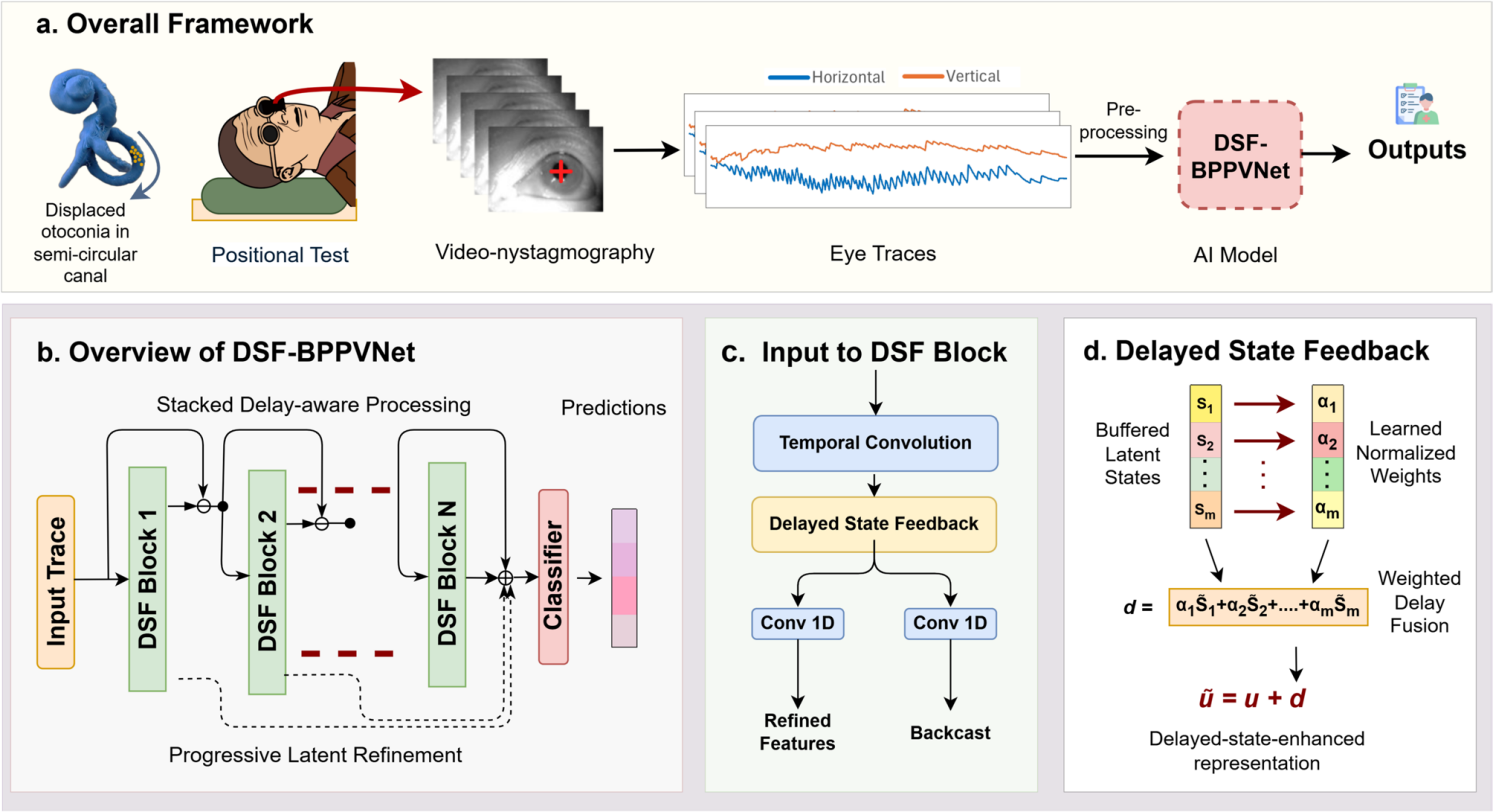
Overview of the proposed DSF-BPPVNet framework. (**a**) Pipeline for classification from VNG, in which traces extracted are provided to DSF-BPPVNet to produce the output class label. (**b**) Overall DSF-BPPVNet architecture, comprising stacked delayed-state feedback blocks. (**c**) Structure of an individual block, combining temporal convolution, delayed-state feedback, and residual refinement. (**d**) Delayed-state feedback mechanism, where buffered latent representations are combined using learned normalized weights to reintroduce delayed information into the current representation.

## Methods

### Problem formulation

Let *X* denote a video-nystagmography (VNG) sequence comprising horizontal and vertical eye-position traces, and let *y* denote the corresponding class label. Depending on the task, *y* may represent either a binary BPPV label or a canal subtype category. The objective is to learn a mapping from the multichannel temporal input *X* to the predicted label $\hat{y}$.

Positional nystagmus is a temporally structured signal in which diagnostically relevant evidence may be distributed across separated portions of the trace rather than concentrated within a single local segment. This motivates a representation in which the current latent update can depend not only on the present feature state but also on delayed latent states retained from earlier stages of processing.

### Delayed-state formulation

A standard state-update formulation computes the next representation primarily from the current state. More generally, a delayed-state formulation allows the current update to depend on both the present representation and a set of delayed states:

1 $$\begin{aligned} z^{(l+1)} = \Phi \left( z^{(l)}, z^{(l-\tau _1)}, z^{(l-\tau _2)}, \dots , z^{(l-\tau _M)}; \theta \right) , \end{aligned}$$

where $z^{(l)}$ denotes the latent representation at processing stage *l*, $\tau _1, \dots , \tau _M$ are discrete delays, *M* is the number of delay terms, and $\Phi (\cdot )$ is a learnable nonlinear transformation parameterized by $\theta$. This formulation is useful when informative evidence is distributed across separated parts of the signal and delayed context may help refine the current representation.

In DSF-BPPVNet, this idea is implemented as a discrete delayed-state feedback mechanism operating on latent feature representations within stacked temporal processing blocks, rather than as an explicit continuous-time delay differential equation solver.

### DSF-BPPVNet architecture

We propose DSF-BPPVNet, a delay-aware neural architecture for BPPV classification from VNG sequences. The model combines temporal convolution, delayed-state feedback, and residual refinement within a stacked block design. Temporal convolution extracts local sequence patterns from the eye-movement traces, delayed-state feedback reintroduces selected prior latent representations through learned weighting, and residual refinement supports progressive updating of intermediate features across blocks. Through this design, the network integrates local temporal feature extraction with delayed latent-state reuse and stage-wise representation refinement. The overall framework is illustrated in Figure 1.

Each block first applies temporal convolutional transformations with nonlinear activation and regularization to produce an intermediate hidden representation from the incoming latent signal. In parallel, a residual pathway is maintained so that each block updates the incoming representation rather than replacing it entirely. Let $r^{(b)}$ denote the residual stream associated with block *b*, and let $u^{(b)}$ denote the hidden representation produced after temporal feature extraction.

To make delayed latent information available during feature extraction, each block maintains a fixed-length buffer of prior latent representations:

2 $$\begin{aligned} S^{(b)} = \left\{ s_1^{(b)}, s_2^{(b)}, \dots , s_M^{(b)} \right\} , \end{aligned}$$

where *M* denotes the number of delay slots. A block-specific delay parameter determines the lag spacing used to select buffered states. Buffered representations chosen at successive delay multiples are combined using learned normalized weights:

3 $$\begin{aligned} \alpha _i^{(b)} = \frac{\exp \left( \omega _i^{(b)}\right) }{\sum _{j=1}^{M} \exp \left( \omega _j^{(b)}\right) }, \quad i = 1, \dots , M, \end{aligned}$$

where $\omega _i^{(b)}$ are trainable parameters and $\alpha _i^{(b)}$ are the corresponding normalized coefficients. The delayed-state contribution is then computed as

4 $$\begin{aligned} d^{(b)} = \sum _{i=1}^{M} \alpha _i^{(b)} \, \tilde{s}_i^{(b)}, \end{aligned}$$

where $\tilde{s}_i^{(b)}$ denotes the buffered representation selected at the *i*-th delay multiple determined by the block-specific delay parameter. This delayed contribution is added to the current hidden representation:

5 $$\begin{aligned} \tilde{u}^{(b)} = u^{(b)} + d^{(b)}, \end{aligned}$$

This operation reintroduces delayed latent information into the current block output using a learned mixture over buffered states, rather than relying only on the present convolutional features. After fusion, the current latent representation is inserted into the buffer and the oldest buffered representation is discarded, enabling delayed information to be carried forward across successive stages of processing.

From the delayed-state-enhanced representation $\tilde{u}^{(b)}$, each block produces two transformed outputs. The first is a backcast term,

6 $$\begin{aligned} b^{(b)} = \rho _{\text {back}}\left( \tilde{u}^{(b)}\right) , \end{aligned}$$

which is subtracted from the residual pathway to generate the input to the next block:

7 $$\begin{aligned} z^{(b+1)} = r^{(b)} - b^{(b)}, \end{aligned}$$

The second is a refined feature representation,

8 $$\begin{aligned} f^{(b)} = \rho _{\text {feat}}\left( \tilde{u}^{(b)}\right) , \end{aligned}$$

which represents the transformed output of the current block. This design separates the role of residual updating from feature refinement. The residual stream provides a pathway for information to be passed forward across blocks, while the refined feature stream captures the transformed representation produced after delayed-state fusion. Across the stacked architecture, this backcast-style update is intended to support progressive refinement of intermediate representations. This residual updating strategy is conceptually related to the backcast refinement used in N-BEATS^22^, although here it is adapted to sequential representation learning for VNG classification rather than forecasting.

After the final temporal processing block, the refined feature representation is aggregated over time to obtain a sequence-level summary, which is then mapped to the output by the final classification layer. The dimensionality of the output layer is task-dependent and is set according to the classification target.

## Experimental protocol

### Dataset

**Fig. 2:**
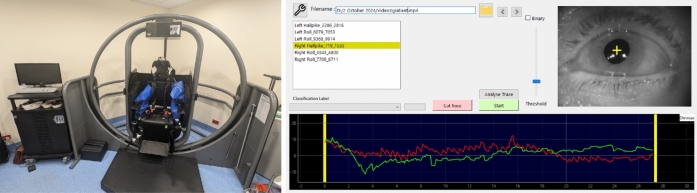
Data acquisition and annotation using the Epley Omniax System (EOS) (left) and OmniVNG software (right).

The dataset consisted of 3,111 2D eye-position traces from 705 patients tested using the Epley Omniax System (EOS) (Figure 2, left), a motorized two-axis rotator chair integrated with real-time VOG. Of these, 1,027 traces were labeled as BPPV and 2,084 as normal/non-BPPV under the study annotation protocol. The BPPV subset comprised 856 posterior-canal cases, 142 horizontal-canal cases, and 29 anterior-canal cases. The normal class comprised traces in which no positional nystagmus was identified during the recorded maneuvers. This group included non-diagnostic eye movements observed during testing, such as movements commonly seen when visual fixation is removed; we elected to retain such recordings when expert review did not indicate BPPV-related positional nystagmus. Recordings were acquired at a public outpatient facility using infrared monocular video-oculography in darkness to eliminate visual fixation. Eye movements were recorded at 30 Hz, and horizontal calibration was performed prior to each test. The dataset included recordings from four standardized diagnostic maneuvers: left Hallpike, right Hallpike, left roll, and right roll. Both horizontal and vertical eye-movement traces were used for analysis; torsional eye-movement information was not available. Raw horizontal and vertical eye-position traces were normalized using z-score normalization to reduce amplitude variability across recordings and mitigate the effect of transient artifacts. The dataset was annotated by a neuro-otologist clinician and a neuro-otologist nurse using the OmniVNG software (Figure 2, right).

### Experiment setup

We considered two evaluation settings: a non-patient-disjoint setting and a patient-independent setting. In the non-patient-disjoint setting, traces were randomly partitioned without enforcing patient-level separation. Within each training fold, 10% of the training data was set aside as a validation set using stratified sampling for model selection and early stopping. In the patient-independent setting, patients were partitioned into disjoint training, validation, and test sets such that no patient contributed traces to more than one split. Class distribution was preserved across folds by stratifying at the patient level. As in the previous setting, 10% of the training patients were allocated to the validation split.

For consistency, all baseline models were re-implemented within the same PyTorch framework and evaluated using the same 5-fold cross-validation splits as DSF-BPPVNet. All models were trained for up to 400 epochs using the Adam optimizer with a learning rate of $1 \times 10^{-3}$. For binary classification, training used binary cross-entropy with logits and early stopping based on validation accuracy with a patience of 100 epochs. For the 4-class subtype experiment, training used multiclass cross-entropy loss and early stopping based on validation macro F1 with the same patience. Evaluation was based on accuracy, precision, recall, and F1-score.

### Hyperparameter selection

To determine the DSF-BPPVNet configuration, we performed a randomized hyperparameter search across 50 settings using 5-fold stratified cross-validation in the non-patient-disjoint setting. The search covered architectural, temporal, and optimization parameters, including the number of temporal convolutional blocks, number of delay states, initial delay stride, convolutional kernel width, pooling strategy, dropout, and learning rate. The delay stride was treated as a learnable parameter in all configurations. The final configuration selected for subsequent experiments used four temporal convolutional blocks, six delay states, a kernel size of 5, an initial delay stride of 6.0, average pooling, dropout of 0.5, and a learning rate of 0.001.

For the baseline models, hyperparameters were selected by randomized search: the 1D-CNN used 64 filters with kernel size 15, the BiLSTM used two layers with a hidden size of 64 per direction, and the Transformer used two encoder layers, four attention heads, and a model dimension of 64.

## Results

### Overall performance

To evaluate the proposed DSF-BPPVNet, we benchmarked its performance against three established deep learning baselines: a 1D-CNN, a Bidirectional LSTM (BiLSTM), and a Transformer encoder. Table 1 summarizes model performance in the patient-independent setting. Across the four models evaluated, DSF-BPPVNet achieved the strongest overall performance, with the highest mean F1-score of 0.819 ± 0.020 and the highest mean recall of 0.808 ± 0.044, together with the highest mean accuracy of 87.88 ± 0.84%. The strongest baseline in this setting was the 1D-CNN, which achieved an F1-score of 0.779 ± 0.097 and a recall of 0.723 ± 0.132. Although the 1D-CNN attained the highest mean precision, 0.855 ± 0.097, its lower recall indicates a less balanced performance than DSF-BPPVNet. The BiLSTM and Transformer baselines yielded lower mean F1-scores of 0.686 ± 0.085 and 0.667 ± 0.051, respectively.

**Table 1:** Overall comparison of patient-independent performance for BPPV classification. Values are mean ± SD across 5 folds.

**Model**	**Accuracy (%)**	**Precision**	**Recall**	**F1**
BiLSTM	79.68 ± 5.24	0.717 ± 0.105	0.660 ± 0.089	0.686 ± 0.085
1D-CNN	86.23 ± 6.01	**0.855** ± **0.097**	0.723 ± 0.132	0.779 ± 0.097
Transformer	78.58 ± 1.36	0.707 ± 0.081	0.647 ± 0.133	0.667 ± 0.051
**DSF-BPPVNet**	**87.88** ± **0.84**	0.834 ± 0.036	**0.808 **± **0.044**	**0.819** ± **0.020**

**Table 2:** Overall comparison of non-patient-disjoint performance for BPPV classification. Values are mean ± SD across 5 folds.

**Model**	**Accuracy (%)**	**Precision**	**Recall**	**F1**
BiLSTM	81.71 ± 3.51	0.748 ± 0.064	0.693 ± 0.071	0.718 ± 0.058
1D-CNN	88.55 ± 1.60	**0.886** ± **0.050**	0.760 ± 0.053	0.817 ± 0.028
Transformer	78.91 ± 2.07	0.706 ± 0.047	0.649 ± 0.108	0.673 ± 0.053
**DSF-BPPVNet**	**89.38 **± **1.20**	0.880 ± 0.025	**0.795** ± **0.057**	**0.834** ± **0.024**

Table 2 reports results in the non-patient-disjoint setting. DSF-BPPVNet again achieved the highest mean F1-score, 0.834 ± 0.024, and the highest mean recall, 0.795 ± 0.057, while also attaining the highest mean accuracy, 89.38 ± 1.20%. The 1D-CNN remained the strongest baseline, with an F1-score of 0.817 ± 0.028. As in the patient-independent setting, the performance of DSF-BPPVNet was characterized by a more favorable balance between precision and recall than the comparator models.

**Fig. 3:**
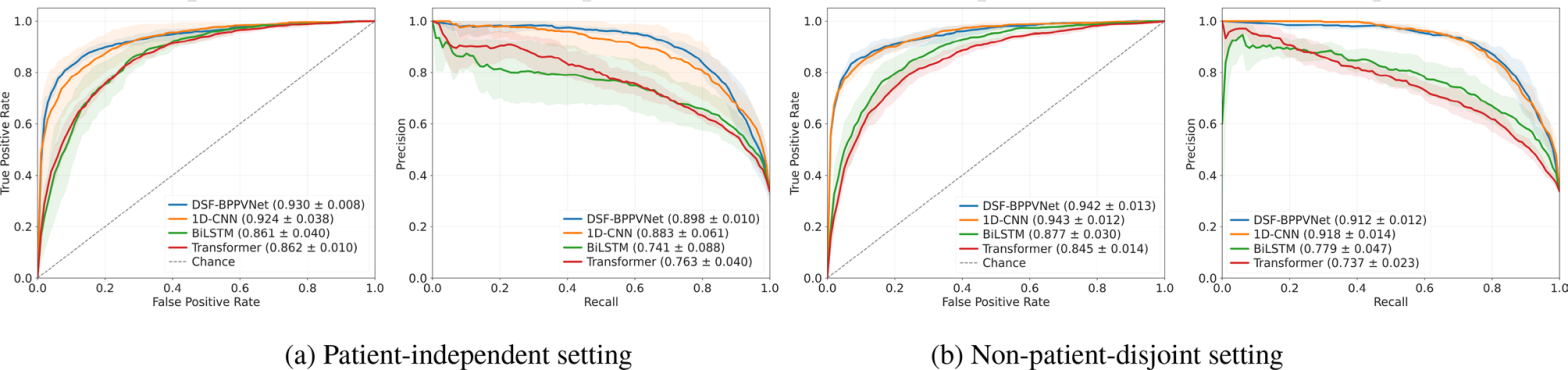
Receiver Operating Characteristic (ROC) and Precision–Recall (PR) curves across 5-fold cross-validation for DSF-BPPVNet and baseline models. The two left panels correspond to the patient-independent setting, and the two right panels correspond to the non-patient-disjoint setting.

The discriminative behavior of the models is further illustrated by the mean Precision–Recall and Receiver Operating Characteristic curves in Figure 3. In the patient-independent setting shown in Figure 3a, DSF-BPPVNet achieved the highest mean ROC-AUC, 0.930 ± 0.008, and the highest mean average precision, 0.898 ± 0.010, exceeding all baselines, including the 1D-CNN with ROC-AUC of 0.924 ± 0.038 and mAP of 0.883 ± 0.061. In addition to its higher mean performance, DSF-BPPVNet showed narrower variability bands across folds, indicating more stable behavior under cross-validation. A similar pattern was observed in the non-patient-disjoint setting shown in Figure 3b, where DSF-BPPVNet again showed the strongest overall Precision–Recall and ROC characteristics.

### Ablation study

To evaluate the contributions of delayed-state feedback and residual refinement, we compared four model variants in the non-patient-disjoint setting, as summarized in Table 3.

The baseline configuration without delayed-state feedback or residual refinement achieved the lowest F1-score, 0.805 ± 0.028. Adding residual refinement alone increased the F1-score to 0.820 ± 0.022, while adding delayed-state feedback alone increased it to 0.825 ± 0.015. The full DSF-BPPVNet model, which includes both components, achieved the highest F1-score, 0.834 ± 0.024. These results suggest that both delayed-state feedback and residual refinement contribute positively to performance. Each component improved upon the baseline when introduced individually, and the best overall result was obtained when both were used together. This pattern is consistent with the two components providing complementary benefits within the full architecture.

**Table 3:** Effect of delayed-state feedback and residual refinement in DSF-BPPVNet in the non-patient-disjoint setting. Values are mean ± SD across 5 folds.

**DSF**	**Refinement**	**Accuracy (%)**	**Precision**	**Recall**	**F1**
$\times$	$\times$	87.79 ± 1.39	0.871 ± 0.023	0.750 ± 0.053	0.805 ± 0.028
$\checkmark$	$\times$	88.55 ± 1.16	0.856 ± 0.052	0.801 ± 0.042	0.825 ± 0.015
$\times$	$\checkmark$	88.30 ± 1.36	0.851 ± 0.039	0.794 ± 0.042	0.820 ± 0.022
$\checkmark$	$\checkmark$	89.38 ± 1.20	0.880 ± 0.025	0.795 ± 0.057	0.834 ± 0.024

### Subtype analysis

We further evaluated DSF-BPPVNet on a more granular 4-class subtype classification task in the patient-independent setting, with classes defined as Normal, PC-BPPV, HC-BPPV, and AC-BPPV. This task is substantially more challenging because of severe class imbalance, with HC-BPPV and AC-BPPV accounting for only 4.6% and 0.9% of the total traces, respectively. DSF-BPPVNet was first trained directly on the 4-class task. We used a Weighted Random Sampler together with a standard cross-entropy loss, as this combination provided the most stable performance during model selection. The aggregated 5-fold confusion matrix for the selected model is shown in Table 4.

To further examine the low AC-BPPV F1-score, we performed a post-hoc one-vs-rest analysis using the saved 4-class model. For each subtype, a probability threshold maximizing the F1-score was selected on the validation set and then applied to the corresponding test-set probabilities. The resulting subtype-specific performance is summarized in Table 5.

The OvR results provide additional insight into class-specific behavior. PC-BPPV was the best-performing subtype, with an F1-score of 0.704 ± 0.066, indicating that the model was able to identify this class with comparatively good precision and recall. HC-BPPV showed lower and more variable performance, with an F1-score of 0.324 ± 0.086. AC-BPPV remained the most difficult class, with a low F1-score of 0.056 ± 0.048 and PR-AUC of 0.048 ± 0.040, despite a ROC-AUC of 0.727 ± 0.038. This combination suggests that, although the model captures some ranking information for AC-BPPV, its subtype discrimination remains weak at practically useful thresholds under the present degree of class imbalance. Overall, these findings indicate that subtype-level performance is strongly influenced by class prevalence and separability, particularly for the rare AC-BPPV class.

**Table 4:** Aggregated 5-fold confusion matrix of the proposed approach.

**True Label**	**Predicted Label**			
	Normal	PC-BPPV	HC-BPPV	AC-BPPV
Normal	1745	200	67	72
PC-BPPV	154	619	62	21
HC-BPPV	25	45	68	4
AC-BPPV	16	9	1	3

**Table 5:** Post-hoc one-vs-rest (OvR) subtype classification performance. Values are mean ± SD across 5 folds.

**Class**	**Precision**	**Recall**	**F1-score**	**PR-AUC**	**ROC-AUC**
PC-BPPV	0.705 ± 0.060	0.709 ± 0.094	0.704 ± 0.066	0.775 ± 0.080	0.881 ± 0.028
HC-BPPV	0.512 ± 0.346	0.435 ± 0.266	0.324 ± 0.086	0.385 ± 0.097	0.777 ± 0.027
AC-BPPV	0.045 ± 0.046	0.247 ± 0.150	0.056 ± 0.048	0.048 ± 0.040	0.727 ± 0.038

### Explainable AI

To better understand the prediction behavior of DSF-BPPVNet, we conducted an explainable AI analysis focusing on both input attribution patterns and the internal behavior of the delayed-state feedback component. Two aspects were examined: input attribution behavior, assessed quantitatively using aggregated Input $\times$ Gradient scores and qualitatively using multiple attribution methods, and the behavior of the delayed-state feedback mechanism, assessed through analysis of the learned delay-weight distributions.

**Fig. 4:**
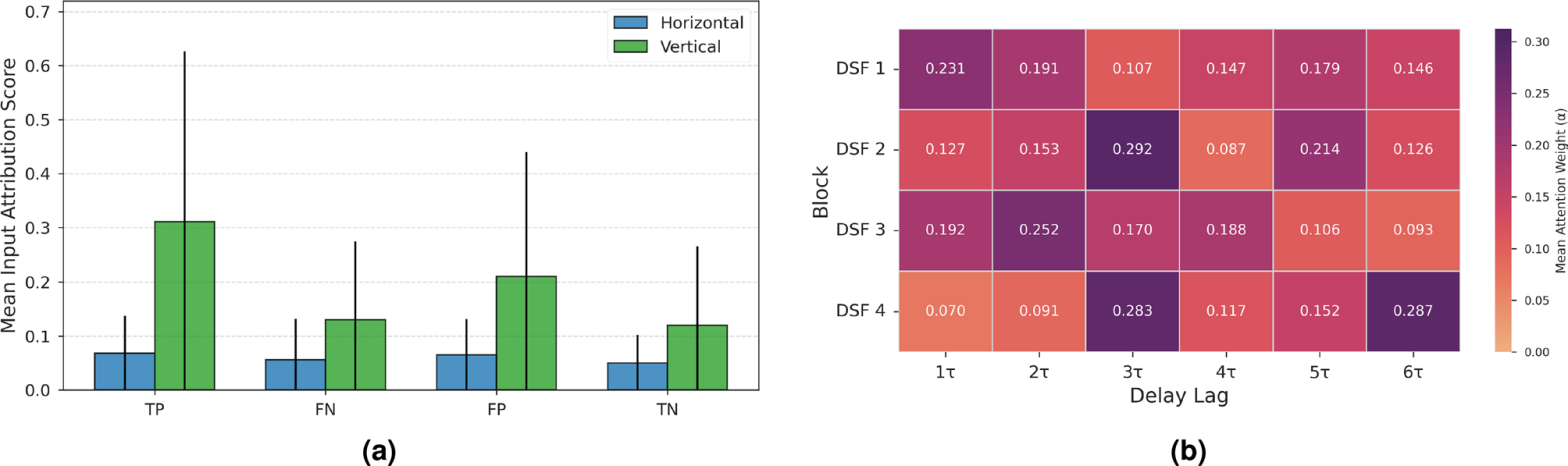
Analysis of DSF-BPPVNet’s learned behavior. (**a**) Aggregated Input $\times$ Gradient attribution scores for horizontal and vertical channels, computed across all samples for each prediction outcome. (**b**) Mean learned delay weights ($\alpha _i$) across blocks and delays, averaged over true-positive cases.

The aggregated mean input attribution scores for the horizontal and vertical channels across the four prediction outcomes are shown in Figure 4a. These results reveal distinct attribution patterns for each outcome and provide insight into the model’s channel-level emphasis. In true positive cases, the model placed substantially greater attribution on the vertical component, with a mean attribution of $0.311 \pm 0.315$, compared with $0.068 \pm 0.069$ for the horizontal component. This pattern suggests that the model places greater emphasis on the vertical channel in correctly identified positive cases. It is also consistent with the predominance of PC-BPPV in the dataset, for which vertical nystagmus is clinically prominent. The analyses of false negatives, false positives, and true negatives show a broadly similar channel-level pattern, suggesting that variation in vertical-channel emphasis is associated with differences in prediction outcome.

To examine the internal behavior of the delayed-state feedback mechanism across processing stages, we analyzed the learned delay weights $\alpha _i$ assigned to each of the six delay indices. Figure 4b presents a heatmap of these weights, averaged across true positive samples from the 5-fold cross-validation, for each of the four convolutional blocks. The weights are relatively distributed across multiple delay lags in the earlier blocks, whereas later blocks assign greater relative emphasis to a smaller subset of delays. In particular, Blocks 2 and 4 assign the highest mean weights to the third delay lag, with average values of 0.292 and 0.283, respectively, while Block 3 places greater weight on the second delay lag, 0.252, and Block 4 also assigns substantial weight to the longest delay, 0.287. These results indicate that the relative contribution of delayed states varies across blocks and suggest that later processing stages place greater emphasis on selected delayed representations.

**Fig. 5:**
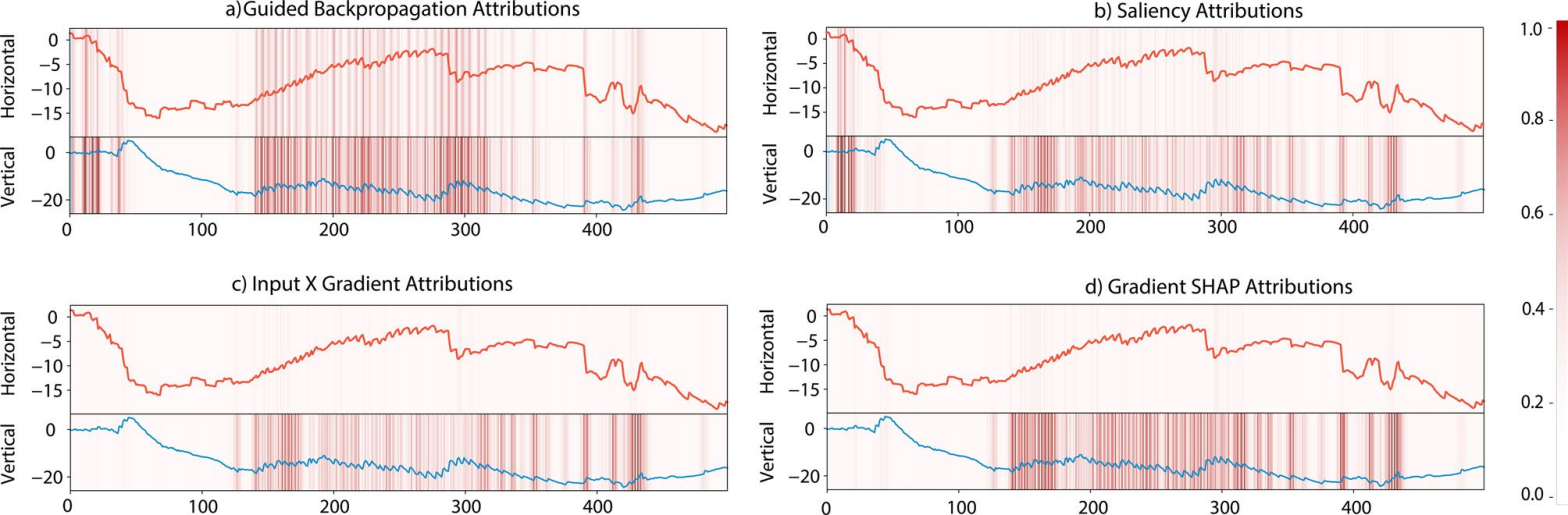
Feature attributions for the horizontal and vertical components of the input traces, visualized using (**a**) Guided Backpropagation, (**b**) Saliency, (**c**) Input $\times$ Gradient, and (**d**) Gradient SHAP methods. Here, the red region represents the magnitude of attribution.

These aggregated findings are further illustrated in the qualitative analysis of a representative true positive sample shown in Figure 5. The figure presents attribution maps generated using four XAI methods: Guided Backpropagation, Saliency, Input $\times$ Gradient, and Gradient SHAP. Across methods, the vertical component shows stronger and more spatially concentrated attribution, particularly during periods of rapid change in the sequence. This qualitative pattern is consistent with the vertical-channel dominance observed in the aggregated analysis in Figure 4a. The attributed regions are also concentrated around portions of the trace corresponding to pronounced temporal transitions, including the apparent onset and decay of nystagmus activity. Taken together, these findings suggest that DSF-BPPVNet is influenced by temporally localized regions of the trace, particularly in the vertical channel.

## Discussion

We developed DSF-BPPVNet, a neural architecture that combines temporal convolution, delayed-state feedback, and residual refinement for classification of BPPV from eye-movement traces. In internal evaluation, DSF-BPPVNet outperformed the evaluated deep-learning baselines in both the patient-independent and non-patient-disjoint settings. The principal improvement was a more favorable precision–recall balance, driven primarily by higher recall while maintaining competitive precision. Within this cohort, these findings indicate that preservation of temporal structure is important for BPPV classification from VNG recordings.

A plausible explanation for this performance is the model’s ability to carry forward and reuse temporally separated information. By incorporating delayed-state feedback, the model reuses information from earlier latent states rather than relying solely on local receptive fields or fully global interactions. This design preserves temporally separated information in eye-movement traces that may be relevant for BPPV classification. In contrast to feature-based methods^11,23,24^ and feature-augmented models^25,26^ that depend on manually derived descriptors such as slow-phase velocity, onset latency, or nystagmus duration, DSF-BPPVNet learns directly from recorded 2D eye-position trajectories. This provides a complementary approach that reduces dependence on hand-engineered features while maintaining sensitivity to temporal structure in the input signal.

The temporal structure of positional nystagmus is clinically informative, but it should not be interpreted as following a single canonical waveform. In clinical practice, its observed time course may vary across patients and BPPV variants and is influenced by interacting factors including endolymph–cupula mechanics, otoconial sedimentation, canal orientation relative to gravity, and the position of debris within the canal^6,27^. These biomechanical determinants help explain clinically observed variation in onset latency, peak intensity, and decay behavior^5^. Accordingly, DSF-BPPVNet is intended to preserve and integrate temporally separated information in the recorded signal rather than to represent a single canonical waveform. Because the network operates directly on eye-position traces rather than explicitly derived slow-phase velocity signals, the learned representation may reflect a combination of slow-phase dynamics, fast-phase reset structure, and broader waveform morphology.

The explainability and ablation analyses support the internal consistency of this design. Delay-weight distributions and attribution maps indicate that deeper blocks make use of non-adjacent temporal context, consistent with the intended function of delayed-state feedback. The ablation results further show that both delayed-state feedback and residual refinement contributed to its performance, with the full model yielding the strongest results. Together, these findings suggest that the observed gains are linked to the proposed architectural components rather than to model scale alone.

Several considerations are important when interpreting these findings. All experiments were conducted on a single-center dataset. Although patient-independent evaluation provides a more rigorous estimate of internal performance, external validation across other devices and acquisition settings will still be necessary to establish broader robustness. In addition, because the present dataset was acquired using the Epley Omniax system, the extent to which these findings transfer to other VNG platforms or bedside VOG recordings remains to be established through external validation. A related consideration is that the non-patient-disjoint setting permits traces from the same patient to appear across splits. Although the difference between the two evaluation settings was modest in the present study, the patient-independent setting provides the more rigorous estimate of internal generalization because it removes subject overlap between training and test data.

The present model operates on 2D eye-movement traces and does not incorporate torsional information. Although vertical eye-movement information can still be informative for distinguishing some vertical-canal patterns, including posterior-canal versus anterior-canal BPPV, the absence of torsional information reduces the completeness of the motion representation and may limit lateralization and finer subtype characterization. This is particularly relevant because canal-specific diagnosis in clinical practice depends on the observed pattern of nystagmus across maneuvers and its relation to canal orientation^6^. The current results therefore support the feasibility of the model for classification from 2D VNG traces, while highlighting the potential value of richer motion representations in future work.

Beyond these representational limitations, the binary task considered here distinguishes BPPV from recordings labeled as normal, but does not encompass the full range of clinically relevant positional disorders. In clinical practice, an important diagnostic challenge is distinguishing benign positional nystagmus from central positional nystagmus and other non-BPPV causes of positional vertigo, including vestibular migraine and cerebellar disorders^4,12^. Extension to such settings, together with finer-grained outputs such as canal- and side-localization and differentiation between canalolithiasis and cupulolithiasis^28^, will be an important next step in assessing clinical applicability more broadly.

Subtype performance should also be interpreted in the context of substantial class imbalance. The dataset was dominated by posterior-canal BPPV, whereas horizontal-canal and especially anterior-canal cases were much less frequent. The attribution analysis suggested predominant reliance on the vertical channel, which may be consistent with stronger separation of posterior-canal cases but could also be associated with weaker performance for anterior-canal BPPV. More balanced datasets, ideally incorporating 3D eye-movement recordings, will be valuable for refining subtype-level classification and for establishing whether these findings generalize more reliably across less prevalent canal subtypes.

## Conclusion

This study presented DSF-BPPVNet, a delay-aware neural architecture for BPPV classification from VNG traces. The model combines temporal convolution, delayed-state feedback, and residual refinement to support classification from temporally structured eye-movement traces. Across the evaluated baselines, DSF-BPPVNet achieved the strongest overall performance in the patient-independent setting and showed similar behavior in the non-patient-disjoint setting. Additional ablation, subtype, and explainability analyses helped characterize the contribution of the proposed components and the model’s prediction behavior. Future work should focus on external multi-site validation, more robust evaluation of rare subtype performance, and extension of the framework to broader vestibular classification tasks.

## Data Availability

Data used in the study is not publicly available however access to the data and code will be made available on request.
